# Heat Stroke with Status Epilepticus Secondary to Posterior Reversible Encephalopathy Syndrome (PRES)

**DOI:** 10.1155/2018/3597474

**Published:** 2018-06-07

**Authors:** Y. H. Koh

**Affiliations:** Department of Neurology, Singapore General Hospital, Singapore

## Abstract

Heat stroke is a life threatening, multisystem disorder characterized by severe hyperthermia (core body temperature > 41.1°C) with central nervous system dysfunction and/or other end organ damage. Neurological complications, such as disturbances of consciousness, convulsion, profound mental change, disorientation, or even prolonged coma, were present in almost all cases of exertional heat stroke (EHS). We present a case of EHS with severe rhabdomyolysis and acute oliguric kidney injury in a 20-year-old healthy marathon runner, who developed status epilepticus on Day 4 of his admission. The patient was managed in ICU with renal replacement therapy and aggressive seizure control. He made a full recovery after 2 weeks of ICU stay. Diagnosis of EHS with posterior reversible encephalopathy syndrome (PRES) secondary to acute kidney injury was made.

## 1. Case Report

A 20-year-old fit and healthy man presented with sudden collapse after running for 3 hours under the hot sun (ambient temperature 39°C) during a marathon competition. After finishing his run for 35 km, he felt unwell and collapsed. He was brought to the hospital immediately. No measures were taken to lower his temperature during the 30-minute transferral to the hospital. Upon arrival to the hospital, he was confused (Glasgow coma scale: 13/15, E4M5V4). His blood pressure was 100/60 mmHg, heart rate 120/min, rectal temperature 42.2°C (axillary temperature 41.5°C). He was given 2 L of intravenous normal saline and rapidly cooled with ice pillow and ice water-soaked towel. He was then transferred to ICU for close monitoring. His rectal temperature was brought down to 39°C (axillary temperature 38.5°C) in an hour time. He was then transferred to ICU.

His cerebral CT scan, electrocardiogram, chest X-ray, and bedside echocardiogram were unremarkable. Laboratory results was shown in Table 1.

He remained oliguric (urine output < 10 ml/hour) despite initial fluid resuscitation. Hemodialysis was commenced on Day 1 of admission. On Day 3 of admission, his arterial blood pH, renal panel, and electrolytes were all normalized and he had regained urine output (urine output 10–20 ml/hour). He was fully conscious and alert (GCS 15/15).

Unfortunately, on Day 4 of admission, he started to complain of severe headache followed by 6 episodes of generalized tonic-clonic seizures, each episode lasting for 1-2 minutes, in a one-hour time. There was no regaining of consciousness in between seizures. The episodes were associated with drooling of saliva, tongue biting, and postictal drowsiness. He was intubated and started on intravenous midazolam infusion, intravenous phenytoin, and intravenous levetiracetam.

**Figure 1:**
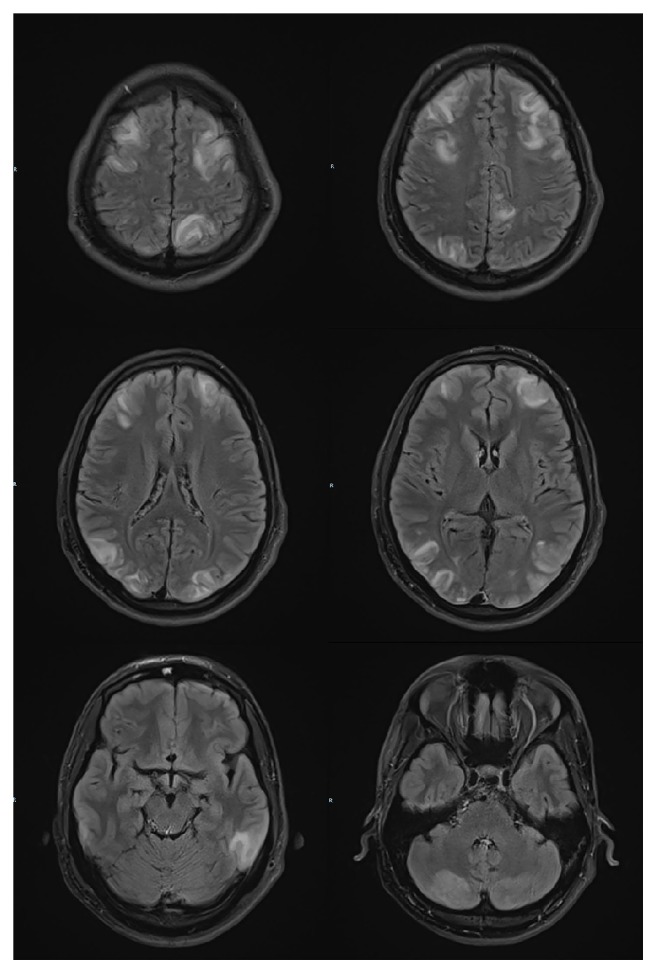
T2 FLAIR sequence. MRI brain (done on Day 4 of heat stroke) showed widespread T2 FLAIR hyperintensities over both cerebral and cerebellar hemispheres, predominantly the cortex and the subcortical white matter.

Electroencephalogram done on the same day showed generalized continuous slowing. However, no epileptic activity was observed during the bedside EEG monitoring. MRI brain done on the same day (Figure 1) showed bilateral, symmetrical vasogenic oedema involving both frontal and posterior parieto-occipital, cerebellar hemispheres, and predominantly the cortex and subcortical white matter regions. DWI and ADC sequence showed no area of restricted diffusion. The finding was consistent with posterior reversible encephalopathy syndrome (PRES).

He had another 2 episodes of generalized tonic-clonic seizures on Day 5 of admission that lasted less than 1 minute. Loading dose of intravenous sodium valproate 1 g was added. His repeat blood tests, including renal panel, complete blood count, pH, and electrolytes, were all normal.

On Day 6 of admission, he was able to produce 3 L of urine per day and hemodialysis was stopped. His midazolam infusion was weaning off slowly. He was fit-free since then. On Day 7 of admission, he was extubated and transferred to general ward. His antiepileptics agents were changed to oral form. His seizure was well controlled with oral phenytoin, valproate, and levetiracetam.

**Figure 2:**
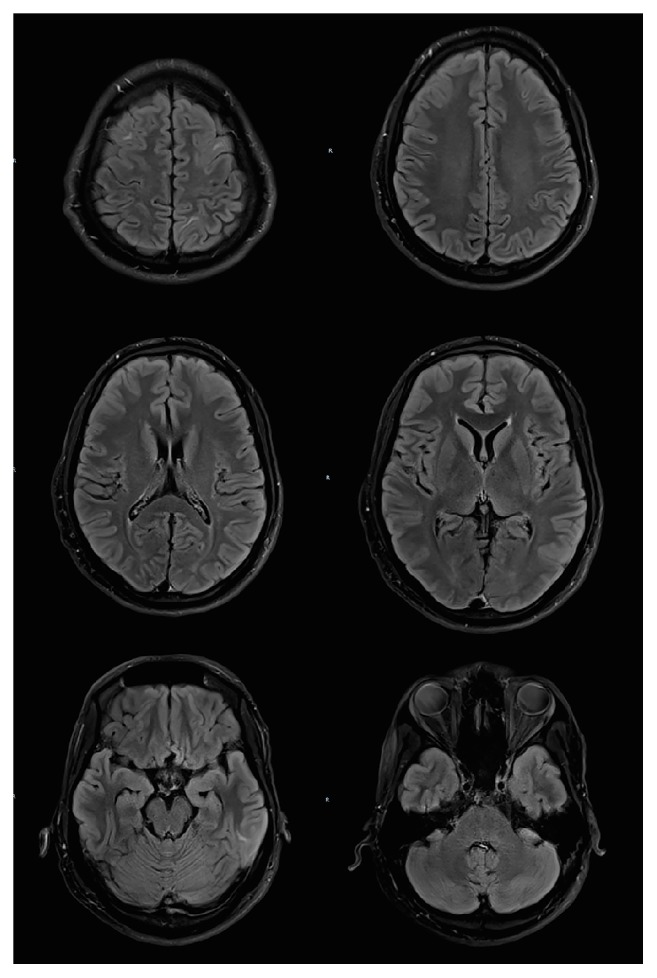
T2 FLAIR sequence. MRI brain done on Day 14 of heat stroke showed near complete resolution of the abnormal cortical and subcortical signal intensity and swelling, compatible with diagnosis of PRES.

A repeat MRI brain done on Day 14 of admission (Figure 2) showed near complete resolution of the previous vasogenic oedema. He was discharged well with oral valproic acid and levetiracetam.

## 2. Discussion

Heat stroke is a spectrum of clinical symptom complexes that occurs when the accumulation of environmental and metabolic heat exceeds the body's heat dissipation rate. It is a life threatening, multisystem disorder characterized by severe hyperthermia (core body temperature > 41.1°C) with central nervous system dysfunction and/or other end organ damage [1].

The pathophysiology of exertional heat stroke is complex and related to regulation of body temperature, which is controlled by anterior hypothalamus [2]. Skin is the major heat-dissipating organ in our body. It dissipates heat to the environment by conduction, evaporation, convection, and radiation. In this case, the patient was running under the hot sun with ambient temperature of 39°C (which was higher than his core body temperature). As a result, conduction of heat from the body to the environment was affected. Moreover, high environmental humidity in Singapore (average percentage of humidity in Singapore is 80%) can definitely affect evaporative cooling mechanism of his body as it decreases the water vapor pressure gradient and shifting of blood from the skin to the active skeletal muscles during strenuous exercise. All these factors eventually lead to failure of heat dissipation and heat stroke in this case.

Rhabdomyolysis, oliguric kidney failure, lactic acidosis, disseminated intravascular coagulation (DIVC), and neurological deficits are well-documented complications in EHS. Renal dysfunction occurs in approximately 30% of heat stroke. Acute renal failure is a well-known cause for PRES. However, this patient's clinical condition has improved after hemodialysis, and his status epilepticus only happened on Day 4 of admission. Recent paper has reported that both the MRI finding and the presentation of PRES can lag behind clinical presentation of acute renal failure [3].

Pathophysiology of heat stroke is not fully understood and still debatable until today. The current model hypothesized that hyperthermia, septicaemia, central nervous system impairment, and cardiovascular failure play an important role in the pathology of heat stroke. There are also studies that advocate that heat stroke is driven by endotoxaemia that triggers the systemic inflammatory response, which further leads to systemic coagulation failure, cell necrosis, multiorgan failures, and clinical presentations of heat stroke [4]. Hence, the occurrence of PRES in this case could be due to the heat stroke and not necessarily due to acute renal failure.

Management of heat stroke can be categorized into 2 stages. First and foremost is early detection, resuscitation, rapid body cooling, and volume depletion. The second stage in management of heat stroke includes treatment of complications, such as rhabdomyolysis, metabolic acidosis, acute renal failure, and seizure [5]. The outcome of heat stroke is related to the degree, the duration, and the severity of hyperthermia [6].

There was a 30-minute delay in the heat stroke treatment in this case as no measures were taken to lower this patient's temperature during his transferral to ED. Rapid lowering of an elevated body temperature is crucial in management of heat stroke. The cooling method should be discontinued once body temperature has reached 38°C. Early detection is crucial as the outcome for EHS is poor when treatment is delayed for more than two hours [7].

In this case, the initial management on his EHS was suboptimal. It took 30 minutes for the patient to reach emergency department where the first measurement of body temperature was taken and cooling measures were commenced. Then It took another hour until the body temperature was reduced. EHS is a medical emergency and it should be treated more aggressively and rapidly.

This patient did show significant improvement after external cooling, rehydration, and renal replacement therapy. His conscious level, urine output, hyperthermia, acute kidney injury, and rhabdomyolysis had almost returned to normal. Unfortunately, the acute kidney injury has resulted in posterior reversible encephalopathy syndrome (PRES). The symptoms of PRES started on Day 4 when he started to have severe headache and status epilepticus.

PRES is a clinicoradiologic entity that refers to a disorder of reversible subcortical vasogenic cerebral oedema in the appropriate clinical context (e.g., uncontrolled hypertension, eclampsia/preeclampsia, autoimmune disorders, immunosuppressant, and renal failure) and presented with acute neurological deficits (e.g., seizure, visual disturbances, headache, and confusion) [8]. PRES primarily affects the subcortical white matter but the cortex is often involved. Brain imaging usually reveals vasogenic oedema in the parieto-occipital region with sparing of calcarine and paramedian parts of the occipital lobes [9], as shown in this case. Involvement of frontal lobes, temporal lobes, cerebellum, basal ganglia, and brainstem can also be seen, usually with concomitant parieto-occipital involvement. Vasogenic oedema of these regions with sparing of parieto-occipital areas, or unilateral vasogenic oedema, should prompt suspicion for an alternative diagnosis.

As the name suggests, the hallmark of PRES is the reversibility of clinical symptoms and radiological findings. It generally has a favorable prognosis, as seen in this patient.

## 3. Conclusion

EHS is a life threatening condition with serious complications. Early recognition, rapid lowering of body temperature, and rehydration are the mainstay of treatment for EHS. PRES is not commonly seen but it can occur in EHS as a result of acute kidney injury. Prognosis for PRES in EHS is good with proper treatment of seizure and other supportive measurements. Last but not least, athletes need to be aware of early signs of EHS and adequate hydration is important during strenuous exercise.

## Figures and Tables

**Table 1:** 

Laboratory Test	Results	Normal Range
*Arterial Blood Gas*		
(i) pH	7.3	(7.35–7.45)
(ii) Base Deficit	15.0 mmol/L	(21–27)
(iii) pCO2	45 mmHg	(35–45)
(iv) pO2	100 mmHg	(75–100)
*Serum Creatine*		
(i) Kinase Level	10625 U/L	(44–201)
*Renal Function *		
(i) Serum Urea	20 mmol/L	(2.7–6.9)
(ii) Serum Creatinine	120 *μ*mol/L	(37–75)
(iii) Serum Potassium	4.5 mmol/L	(3.6–5.0)
(iv) Serum Sodium	146 mmol/L	(136–146)
*Urine Myoglobin*	141 UG/L	(<21)
*Complete Blood Count*	Normal	Normal
*Coagulation Profile*	Normal	Normal
*Liver Function Test*	Normal	Normal
